# Association between Hemoglobin and Hemoglobin A_1c_: A Data-Driven Analysis of Health Checkup Data in Japan

**DOI:** 10.3390/jcm7120539

**Published:** 2018-12-12

**Authors:** Masato Takeuchi, Koji Kawakami

**Affiliations:** Department of Pharmacoepidemiology, Graduate School of Medicine and Public Health, Kyoto University, Kyoto 606-8501, Japan; takeuchi.masato.3c@kyoto-u.ac.jp

**Keywords:** anemia, diabetes mellitus, spline curve, generalized additive mixed model, machine learning

## Abstract

Background: Interpretation of hemoglobin A_1c_ (HbA_1c_) levels may be confounded by spurious results in anemic persons, but its degree is not well-established. Methods: We used an employer-based health insurance database, containing health checkup data and medical claims data; both were linked via a unique identifier of each beneficiary. This study included persons aged 18–75 years who participated in health checkups, with a confirmed or suspected diagnosis of diabetes. The relationship between hemoglobin (Hb) and HbA_1c_ is shown in a spline curve using a machine learning technique accounting for patient factors and within-person correlations. Spline curves were also shown in several sub-populations. Results: Overall, a decreased Hb value was associated with a lower HbA_1c_ value, but the extent differed among populations. In the whole cohort of the type-2 diabetes group (55,420 persons), the curve was generally a plateau in the persons with a Hb value <120–130 g/L. Among the 18,478 persons with HbA_1c_ around 48 mmol/mol, we observed a liner trend. Among the current glucose-lowering medication users (6253 persons), we found a right upward curve. Conclusions: The relationship between Hb and HbA_1c_ may not be straightforward, varying among populations of different clinical interest. Our results indicate that a simple formulation between the Hb and HbA_1c_ values is unlikely.

## 1. Introduction

The number of persons with diabetes is rapidly increasing worldwide, particularly with type-2 diabetes mellitus (T2DM) [1]. The number of persons with diabetes is expected to increase from 425 million in 2018 to 642 million by 2040, with T2DM accounting for more than 90% of these persons [2]. The increase of T2DM is supposed to be associated with an increase in obesity, sedentary lifestyle, and energy-dense diets [3]. Although the data are somewhat inconsistent, the incidence and prevalence of type 1 diabetes mellitus (T1DM) are also reported to be increasing [4].

Hemoglobin A_1c_ (HbA_1c_) is widely used as measure for the diagnosis and management of diabetes [5]. The advantages of HbA_1c_ over other metrics of glucose control monitoring are its convenience for patient and the ease of sample collection; HbA_1c_ sampling can be obtained at any time, requires no patient preparation (e.g., fasting), and is relatively stable at room temperature [6]. However, the HbA_1c_ value may exhibit spurious results influenced by non-glycemic factors including anemia, hemoglobin variants, or chronic illness (e.g., nutrient deficiency, liver failure, and end-stage renal disease) [7]. Among these factors, the degree to which the HbA_1c_ value is confounded by anemia remains unknown. For example, although most of the earlier works found that iron deficiency anemia was related to elevated HbA_1c_ levels [8], the reported degree varied from 3 to 23 mmol/mol [9,10,11,12]. These comparisons were made between different persons with and without iron deficiency anemia, or between the same persons before and after iron replacement. The paucity of the existing knowledge is partly because there has been only a limited number of existing studies to date, with low participant numbers [8]. Also, because of the different definitions used for anemia in these previous studies, the interpretation of the findings may be complicated.

We hypothesized that graphical presentation using continuous values for hemoglobin (Hb) and HbA_1c_ without assumption would be easy-to-interpret. In addition, we assumed that a large number of participants would increase the generalizability. For these backgrounds, the present study sought to graphically describe the associations between the Hb and HbA_1c_ values, using a large-scale regular health checkup data of the Japanese population with or without diabetes.

## 2. Experimental Section

### 2.1. Participants

We used an employer-based health insurance database in Japan, compiled by a commercial data vendor (Japan Medical Data Center, Tokyo, Japan), which included the health records of a total of 4.8 million individuals from nearly 100 insurances [13]. The database included health checkup data and medical claims data, and both of these data were linked via a unique identifier assigned to each beneficiary. From this database, the data vendor extracted persons (1) who had a data record of at least health checkup, and (2) who had at least one claims record of confirmed or suspected diagnosis of diabetes during 2005–2013.

In Japan, the annual health checkup has been recommended for employees since the 1970s, and for all persons aged 40–74 years since 2009. Thus, this study involved the checkup data of (1) employees of all-ages during 2005–2013, as well as of (2) their family members aged 40–74 years during 2009–2013. The health checkup data typically included valuables such as Hb, HbA_1c_, fasting glucose level, body mass index, and self-reported current smoking status, but it did not include the serum ferritin level or reticulocyte count. We also utilized the prescription data and diagnostic records of the claims database.

**Figure 1:**
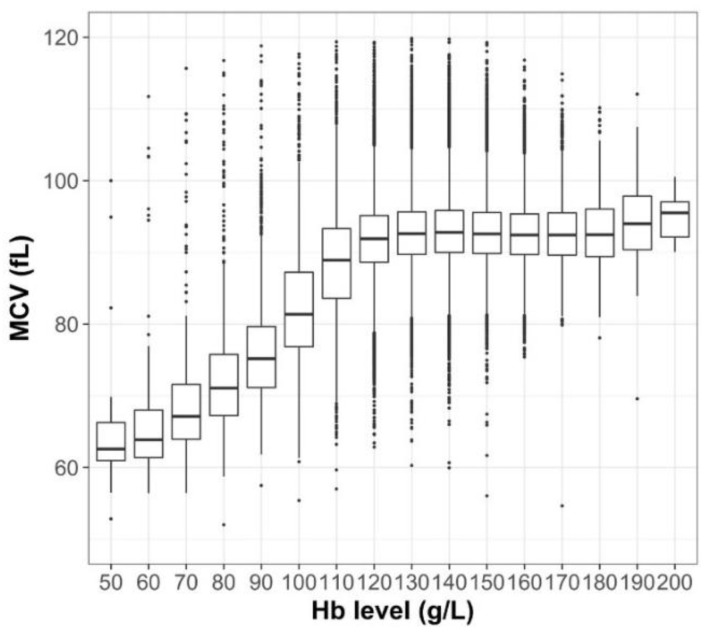
Distribution of mean corpuscular volume, by hemoglobin (Hb) value. The horizontal line in the middle of each box indicated the median, and the top and bottom borders of the box mark the 75th and 25th percentiles, respectively. The whiskers above and below the box mark represent the upper and lower adjacent data, covering >99% of the data range. Outliners are presented in dots. Hb values were grouped after rounding to the nearest integer. MCV, mean corpuscular volume.

From the dataset extracted by the data vendor, we created two separate cohorts for T2DM and T1DM. The T2DM cohort included at least one diagnostic record of T2DM (International Classification of Diseases, 10th Revision (ICD-10) code: E11). In the Japanese health insurance system, records of unconfirmed diagnosis are acceptable for claims purposes (e.g., recorded as “suspected” T2DM); this kind of diagnosis was often made when performing a laboratory examination for persons at risk of developing the condition. This study did not exclude persons with unconfirmed T2DM, so that this cohort represented a mixture of persons with confirmed T2DM and those at risk of T2DM. From this T2DM cohort, we formed three sub-cohorts. First, we identified persons undergoing current treatment who had glucose-lowering agents, within 90 days of a health checkup in an outpatient setting. The second sub-cohort included persons with HbA_1c_ values around a diagnostic threshold of 48 mmol/mol (37–58 mmol/mol). The third cohort comprised persons who received iron-supplementation. We identified persons with an outpatient prescription of iron preparation within 90 days of a health checkup. Because the data of serum ferritin—the standard diagnostic measure of iron deficiency anemia—were lacking in the health checkup data, we assumed that the persons with active iron supplementation represented persons with iron deficiency anemia. We additionally calculated the mean corpuscular volume (MCV) for each person, in order to see how likely it was that the anemia was caused by an iron deficiency in our population; the results are presented in Figure 1. Finally, the criteria for T1DM cohort were as follows: (1) having the diagnostic code for T1DM (ICD-10: E10); (2) receiving insulin maintenance therapy, identified by an insulin prescription record from at least two separate outpatient encounters; and (3) excluding persons with a suspected diagnosis. For the T1DM cohort, a sub-cohort was not created.

### 2.2. Statistical Analysis

The participant characteristics were summarized as descriptive statistics. The relationship between the Hb and HbA_1c_ values was displayed in a spline curve constructed without a pre-specified relationship between the variables (e.g., linearity). We developed spline curves using a general additive mixed effects model adjusting for age, sex, fasting glucose level, body mass index, smoking status, and within-person correlation [14]. Persons with a high Hb or HbA_1c_ level at baseline tended to have high levels of these values in the later checkup tests, and vice versa; a mixed effects model was used to account for these within-person correlations in the repeated measures. Participants without the aforementioned covariates were excluded from the analysis. We created several spline curves in all of the cohorts defined above. Although model computations were done using a high-performance computer with 128 GB available in RAM memory, some computations were terminated because of a memory limit. In such cases, we adequately used a random sampling of the participants (e.g., 20%) for computation; we have confirmed that the graphical relationship between Hb and HbA_1c_ was not affected by these different sampling sets (data not shown).

All of the statistical analyses were done with R statistical software version 3.43 (https://www.r-project.org/) with the use of *mgcv* package version 1.8-23 for computing generalized additive mixed model.

## 3. Results

After excluding 24.9% of the participants (*n* = 18,338) with missing data, this study enrolled 55,420 persons in the T2DM cohort and 598 persons in the T1DM cohort, with a mean of 2.7 checkup records for each person. Table 1 summaries the characteristics of the study cohort.

In our study cohort, there was a trend towards a lower MCV in anemic persons (Figure 1).

**Figure 2:**
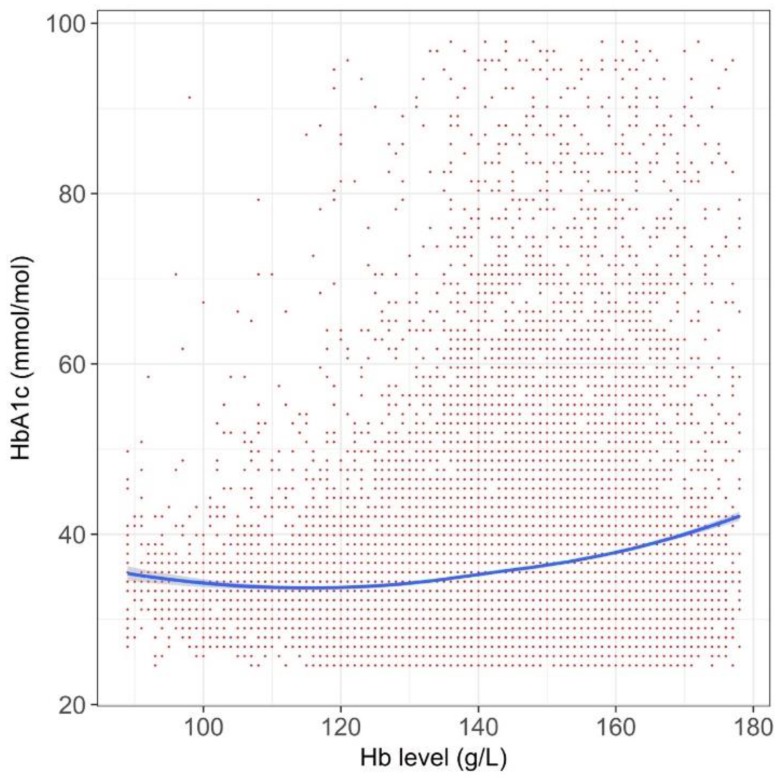
The relationship between Hb and hemoglobin A_1c_ (HbA_1c_) value for the whole cohort (*n* = 55,420). Data are shown in a spline curve with a 95% confidence interval (gray zone). Dots represent the raw data of each person, with overlapping allowed. The range of axis was chosen in order to cover ~99% of the target population.

Overall, a decreased Hb value was associated with a lower HbA_1c_ value, after adjusting the baseline characteristics, but the degree of this trend differed among populations. In the overall T2DM cohort, the relationship between the Hb and HbA_1c_ value was generally a plateau in persons with a Hb value <120–130 g/L, and had a right upward trend above this range (Figure 2).

**Figure 3:**
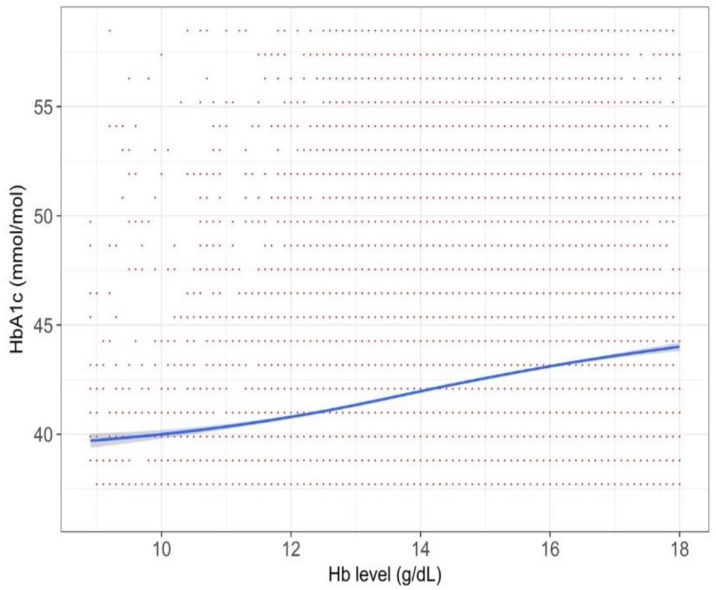
The relationship between Hb and HbA_1c_ value in persons with HbA_1c_ values of around 48 mmol/mol (*n* = 18,478). Graph was created with a 75% random sampling.

For the T2DM sub-cohort with an HbA_1c_ diagnostic threshold close to that of diabetes (i.e., 48 mmol/mol), we noted a liner upward trend (Figure 3).

**Figure 4:**
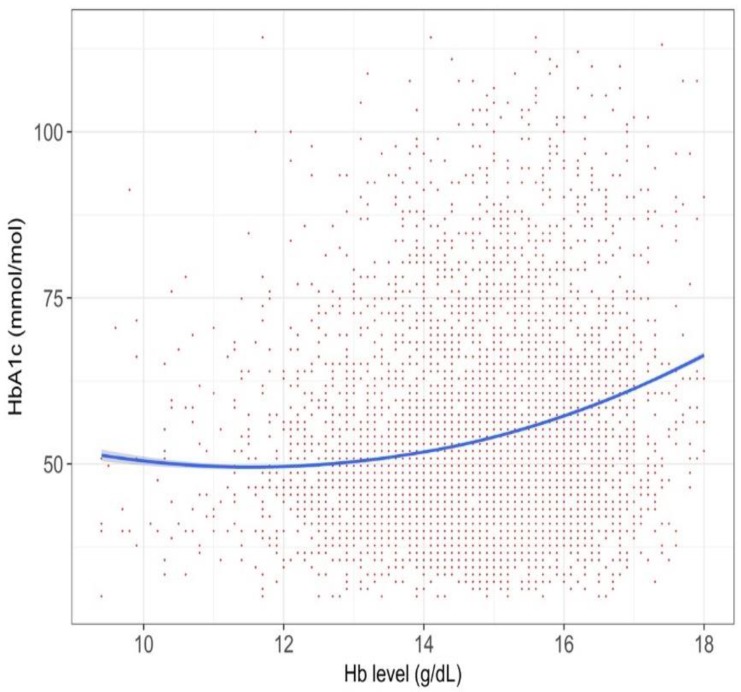
The relationship between Hb and HbA_1c_ values in persons with glucose-lowering medication prescription, within 90 days of their health checkup (*n* = 6253).

In the T2DM sub-cohort that included persons currently taking glucose-lowering medication (6253 persons), we found a global right upper tend (Figure 4).

**Figure 5:**
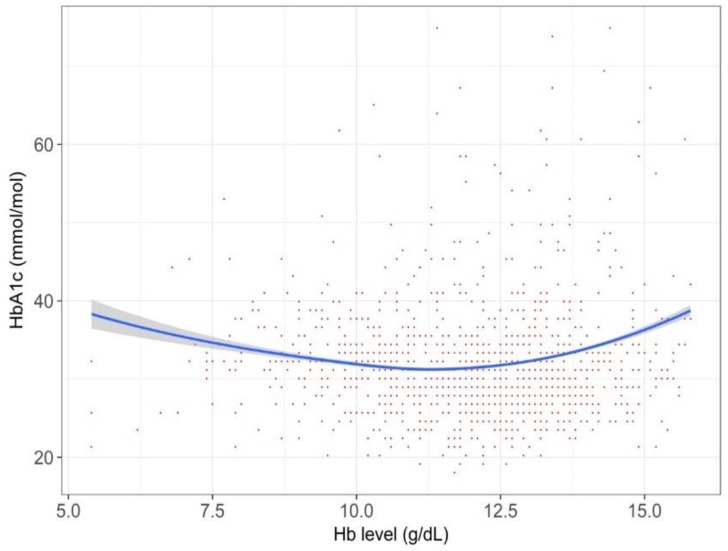
The relationship between Hb and HbA_1c_ values in persons with an iron prescription, within 90 days of their health checkup (*n* = 956).

The relationship in the T2DM sub-cohort taking iron-supplementation (956 persons) showed a U-shaped relationship (Figure 5).

**Figure 6:**
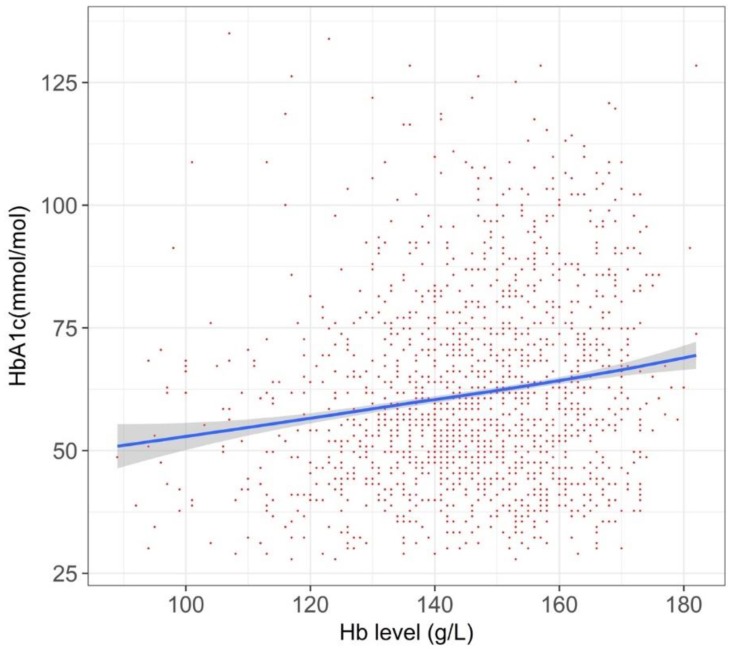
The relationship between Hb and HbA_1c_ values in persons in the T1DM cohort (*n* = 598). T1DM, type 1 diabetes mellitus.

We also found that the relationship between Hb and HbA_1c_ showed a linear trend among 598 persons in the T1DM cohort (Figure 6)

## 4. Discussion

We found that the relationship between Hb and HbA_1c_ was complicated. Overall, the HbA_1c_ values in anemic persons were lower than those in non-anemic persons (i.e., persons with normal or high Hb), but its degree varied among populations. According to a systematic review in 2015, establishing the degree of anemia on the reliability of HbA1c is inconclusive [8]. This review identified 12 relevant studies, but the numbers of patients were typically small in each study. Another caveat of the prior studies was that they compared two groups, divided by a specific cut-off value for anemia. Such a categorization may miss within-group variations [15], and we indeed observed different patterns within the anemic populations (right side of Figure 2, Figure 3, Figure 4, Figure 5 and Figure 6) or within the non-anemic populations (left side of Figure 2, Figure 3, Figure 4, Figure 5 and Figure 6).

The influence of anemia on HbA_1c_ concentration can be cause-specific. For example, iron-deficiency may shift HbA_1c_ slightly upward, whereas other forms of anemia or the recovery phase from iron deficiency anemia are associated with lower HbA_1c_ [16]. We could not determine the cause of anemia in each participant, because of the absence of markers (e.g., ferritin level) in the health checkup data. As shown in Figure 1, a lower MCV in anemic persons suggests that anemia was largely due to the iron-deficiency. However, it is possible that the anemic populations in our study were a mixture of persons suffering from iron-deficiency anemia with or without treatment, and those of other types of anemia. Previous studies focused on the specific types of anemia, such as nutritional-deficiency or menstruation [4], leading to specific results forming a selected but homogenous population at the cost of generalizability. On the hand, in this study, the data from diverse populations lead to generalizability, but we may have overlooked the relationship between anemia and HbA_1c_ values in certain populations.

We observed different response patterns of Hb and HbA_1c_ among different populations, unexpectedly. Different baseline characteristics other than the Hb and HbA_1c_ valuables may be the cause, although we have statistically addressed these potential confounders where possible, by applying a mixed effects model. Our research suggests that it will be challenging to develop a simple correction formula between anemia and HbA_1c_ that is applicable to a universal population. For these reasons, the present study may give warning that treatment decisions based solely on HbA_1c_ measurement without the consideration of other clinical data may lead to overdiagnosis or overtreatment, reinforcing the statement in the recent guideline [9]. This study also suggests that it is difficult to define the optimal threshold of anemia, and to explore the relationship between anemia and HbA_1c._ This implicates that, in future studies, the Hb value should be treated as a continuous value in order to minimize the loss of information by categorization [17].

Our study exhibits an explanatory relationship between the Hb and HbA_1c_ values analyzed by a data-driven approach, rather than by establishing causality, yet our findings are, not fully, but to some extent, consistent with previous researches. Firstly, one recent study found that the degree of iron deficiency anemia influenced the HbA_1c_ level [18], in which severe anemia was associated with a higher HbA_1c_ level. This may explain the phenomena in the left side of Figure 5, showing a left upward trend among persons receiving active treatment of iron supplementation. Secondly, our graphs consistently showed a right upward trend. In polycythemia, the HbA_1c_ may increase because of a longer red blood cell lifespan [19], and the relationship between polycythemia and elevated HbA_1c_ was reported in some literature of rare, congenital hemoglobin variants [20,21]. The right upward trends in the Figures could be explained by such a theoretical ground.

Here, we discuss limitations of our study. First, it is unclear whether our results are applicable to other populations, such as elderly persons or other ethnic groups. For example, among persons with type 1 diabetes, the overestimation of HbA_1c_ levels could occur in black persons compared with white persons, possibly because of racial differences in the hemoglobin glycation [22]. In our study, participants were limited to the Japanese population, and a different response pattern between Hb and HbA_1c_ might found in other ethnic groups. Also, our study population was relatively young. Accordingly, it is unknown whether our findings are replicable in other settings. Second, we could not control the interlaboratory variations of laboratory measures, if any. These limitations highlight the need for more research on this topic.

## 5. Conclusions

We presented the graphical overview on the relationship between Hb and HbA_1c_ from large-scale data in Japan. We found out that to what degree anemia affected the HbA_1c_ was varied among the population, and the relationship is unlikely to be easily formulated nor converted. Our study did not reveal the causality, and the data from the unselected populations might be of limited value for individual-level use; these issues would be an area for future research. Our findings imply that the management of diabetes relying solely on the HbA_1c_ value warrants careful consideration in a subset of populations, such as in persons with anemia.

## Figures and Tables

**Table 1:** Characteristics of the study cohort.

Parameters	All T2DM Participants (*n* = 55,420)	HbA_1c_ around Diagnostic Threashold ^1,2^ (*n* = 18,478)	Current Drug Users ^1,3^ (*n* = 6253)	Iron-Supplement Cohort ^1^ (*n* = 956)	T1DM Cohort (*n* = 598)	Iron-Supplement Cohort (*n* = 956)
Age ^3^ (years)	46.5 (10.7)	51.2 (9.3)	51.4 (9.3)	44.2 (8.3)	48.4 (10.1)	44.2 (8.3)
Sex (male)	59.4%	66.4%	77.4%	17.8%	72.2%	17.8%
BMI ^3^ (kg/m^2^)	23.8 (4.3)	25.3 (4.5)	25.9 (4.7)	22.3 (4.2)	24.7 (4.7)	22.3 (4.2)
Hb ^4^ (g/L)	143 (16)	144 (16)	148 (15)	118 (19)	141 (17)	11.8 (19)
HbA1c ^3 ^(mmol/mol)	35 (11)	41 (5)	52 (16)	33 (9)	63 (21)	33 (9)
fBG ^3^ (mg/dL)	104 (33)	114 (27)	149 (55)	97 (29)	168 (74)	97 (29)
Smoking	32.1%	32.2%	37.9%	21.8%	40.3%	21.8%

^1^: Persons with T2DM; ^2^: 48 mmol/mol in IFCC unit; ^3^: Current glucose-lowering drug users; ^4^: Data are shown as mean with standard deviation; T2DM, type 2 diabetes mellitus; T1DM, type 1 diabetes mellitus; BMI, body mass index; Hb, hemoglobin; HbA1c, hemoglobin A1c; fBG, fasting blood glucose.
